# Supplementary material for: Efficacy and safety of albendazole 400 mg for 30 days compared to single dose of ivermectin in adult patients with low Loa loa microfilaremia: A non-inferiority randomized controlled trial
Source: PLoS Negl Trop Dis. 2025 Jun 20;19(6):e0012383. doi: 10.1371/journal.pntd.0012383 (PMC12212867; doi:10.1371/journal.pntd.0012383)
Supplement: S1 Table — The dataset of the provided results. (PDF) [file pntd.0012383.s001.pdf]

numéro PHYLECOG poids (Kg) taille (Cm) taille (m) IMC traitement administré Mf D0 symptome D0 oedeme D0
ver dans œil D0 reptation D0 prurit D0 Mf D2 symptome D2 oedeme D2 ver dans œil D2 reptation D2 prurit D2 ES D2
asthenie D2 gravité asthenie D2 maux de tete D2 vomissements D2 diarrhee D2 nausee D2 vertige D2 appetit D2
perte d'appetit D2 Mf D7 symptome D7 oedeme D7 ver dans œil D7 reptation D7 prurit D7 ES D7 asthenie D7 gravité
asthenie D7 maux de tete D7 vomissements D7 diarrhee D7 nausee D7 vertige D7 appetit D7 perte d'appetit 7 Mf
D14 symptome D14 oedeme D14 ver dans œil D14 reptation D14 prurit D14 ES D14 asthenie D14 gravité asthenie D14 maux de
tete D14 vomissements D14 diarrhee D14 nausee D14 vertige D14 appetit D14 perte d'appetit 14 Mf D30 symptome D30
oedeme D30 ver dans œil D30 reptation D30 prurit D30 ES D30 asthenie D30 gravité asthenie D30 maux de tete D30
vomissements D30 diarrhee D30 nausee D30 vertige D30 appetit D30 perte d'appetit 30

1 PHY-0022 53,2 158 1,58 21,31 A400 3200 oui non oui oui oui 700 oui non non oui
oui oui oui légère oui non non non oui non non 200 non non non non non oui
oui légère oui non non non non non non 200 non non non non non non non légère
non non non non non non non 100

2 PHY-0024 50,5 173 1,73 16,87 A400 1600 non non non non non 400 non non non non
non non non légère non non non non non non non 700 non non non non non non
non légère non non non non non non non 400 non non non non non non non légère
non non non non non non non 200

3 PHY-0109 63,3 166 1,66 22,97 ivermectine 1300 oui non non oui oui 300 oui non non
non non oui oui légère non non non non non non non 600 non non non non non
oui oui légère oui non non non non non non 200 non non non non non non non
légère non non non non non non non 100 non non non non non non non légère non
non non non non non non

4 PHY-0212 71,1 181 1,81 21,70 A400 1500 non non non non non 500 non non non non
non oui oui légère non non non non non non non 400
200
400 non non non non non non non légère non non non non
non non non

5	PHY-0534		82,1	180	1,8	25,34	A400	1600	oui	non	non	oui	oui	700	oui	non	non	non
	oui	non	non	légère	non	non	non	non	non	non	non	1000	non	non	non	non	non	oui
	oui	légère	non	non	non	non	non	non	non	400	non	non	non	non	non	non	non	légère
	non	non	non	non	non	non	non	400	non	non	non	non	non	non	non	légère	non	non
	non	non	non	non	non													
6	PHY-0544		59,3	155	1,55	24,68	A400	700	oui	non	non	non	oui	800	oui	non	non	non
	oui	non	non	légère	non	non	non	non	non	non	non	500	non	non	non	non	non	non
	non	légère	non	non	non	non	non	non	non	400	non	non	non	non	non	non	non	légère
	non	non	non	non	non	non	non	400										
7	PHY-0560		56,5	153	1,53	24,14	A400	900	oui	non	non	non	oui	1200	oui	non	non	non
	oui	non	non	légère	non	non	non	non	non	non	non	900	non	non	non	non	non	non
	non	légère	non	non	non	non	non	non	non									
8	PHY-0573		63	155	1,55	26,22	ivermectine		500	oui	non	non	non	oui	0	oui	non	non
	non	oui	non	non	légère	non	non	non	non	non	non	non	300	oui	non	non	non	oui
	non	non	légère	non	non	non	non	non	non	non	6	non	non	non	non	non	non	non
	légère	non	non	non	non	non	non	non	0	non	non	non	non	non	non	non	légère	non
	non	non	non	non	non	non												
9	PHY-0637		55,9	158	1,58	22,39	ivermectine		2200	oui	non	non	non	oui	3900			
												800	non	non	non	non	non	oui
	oui	légère	non	non	non	non	non	non	non	300	non	non	non	non	non	non	non	légère
	non	non	non	non	non	non	non	300	non	non	non	non	non	non	non	légère	non	non
	non	non	non	non	non													
10	PHY-0672		86,8	174	1,74	28,67	A400	600	non	non	non	non	non	800	non	non	non	non
	non	non	non	légère	non	non	non	non	non	non	non	100	non	non	non	non	non	non
	non	légère	non	non	non	non	non	non	non	100	non	non	non	non	non	non	non	légère

	non	non	non	non	non	non	non	1100	non	non	non	non	non	non	non	légère	non	non
	non	non	non	non	non													
11	PHY-0674		90,1	173	1,73	30,10	A400	2200	non	non	non	non	non					
											300	non	non	non	non	non	non	non
	légère	non	non	non	non	non	non	non	300	non	non	non	non	non	non	non	légère	non
	non	non	non	non	non	non	200	non	non	non	non	non	non	non	légère	non	non	non
	non	non	non	non														
12	PHY-0675		67,2	177	1,77	21,45	A400	1500	non	non	non	non	non	600	non	non	non	non
	non	non	non	légère	non	non	non	non	non	non	non	200	non	non	non	non	non	non
	non	légère	non	non	non	non	non	non	non	2,00	non	non	non	non	non	non	non	légère
	non	non	non	non	non	non	non	4	non	non	non	non	non	non	non	légère	non	non
	non	non	non	non	non													
13	PHY-0695		67	172	1,72	22,65	A400	2100	non	non	non	non	non	2000	non	non	non	non
	non	oui	oui	légère	non	non	non	non	non	non	non							
14	PHY-0710		54,1	159	1,59	21,40	ivermectine		600	oui	oui	non	non	non	54	oui	oui	non
	non	non	oui	non	légère	non	non	oui	non	non	non	non	5	non	non	non	non	non
	non	non	légère	non	non	non	non	non	non	non	16	non	non	non	non	non	non	non
	légère	non	non	non	non	non	non	non	10	non	non	non	non	non	non	non	légère	non
	non	non	non	non	non	non												
15	PHY-0728		57,2	158	1,58	22,91	ivermectine		600	oui	oui	non	non	non	300	non	non	non
	non	non	non	non	légère	non	non	non	non	non	non	non	16					
										6,4	non	non	non	non	non	non	non	légère
	non	non	non	non	non	non	non	2	non	non	non	non	non	non	non	légère	non	non
	non	non	non	non	non													

16	PHY-0737		65,5	165	1,65	24,06	ivermectine		1000	non	non	non	non	non	200	oui	non	non
	non	oui	non	non	légère	non	non	non	non	non	non	non	1	non	non	non	non	non
	non	non	légère	non	non	non	non	non	non	non	200	non	non	non	non	non	non	non
	légère	non	non	non	non	non	non	non	1	non	non	non	non	non	non	non	légère	non
	non	non	non	non	non	non												
17	PHY-0740		57	164	1,64	21,19	A400	1500	oui	non	non	oui	non	900	non	non	non	non
	non	oui	oui	légère	non	non	non	non	non	non	non	800	non	non	non	non	non	oui
	oui	légère	non	non	non	non	non	non	non	200	non	non	non	non	non	oui	oui	légère
	non	non	non	non	non	non	non	200	non	non	non	non	non	non	non	légère	non	non
	non	non	non	non	non													
18	PHY-0755		74	156	1,56	30,41	ivermectine		2200	oui	non	non	oui	oui	100	oui	non	non
	non	oui	non	non	légère	non	non	non	non	non	non	non	200	oui	non	non	non	oui
	non	non	légère	non	non	non	non	non	non	non	300	non	non	non	non	non	non	non
	légère	non	non	non	non	non	non	non	300	non	non	non	non	non	non	non	légère	non
	non	non	non	non	non	non												
19	PHY-0760		62,1	159	1,59	24,56	A400	500	oui	oui	non	non	non	800	non	non	non	non
	non	non	non	légère	non	non	non	non	non	non	non	200	non	non	non	non	non	non
	non	légère	non	non	non	non	non	non	non	1	non	non	non	non	non	non	non	légère
	non	non	non	non	non	non	non	1	non	non	non	non	non	non	non	légère	non	non
	non	non	non	non	non													
20	PHY-0788		66,7	155	1,55	27,76	ivermectine		1300	oui	non	non	non	oui	900	non	non	non
	non	non	oui	non	légère	oui	non	non	non	oui	non	non	200	non	non	non	non	non
	non	non	légère	non	non	non	non	non	non	non								
21	PHY-0847		76,9	172	1,72	25,99	ivermectine		2000	oui	non	non	non	oui	600	oui	non	non
	non	oui	non	non	légère	non	non	non	non	non	non	non						

22	PHY-0867		52,5	159	1,59	20,77	A400	1200	non	non	non	non	non	700				
											400	non	non	non	non	non	non	non
	légère	non	non	non	non	non	non	non	600	non	non	non	non	non	non	non	légère	non
	non	non	non	non	non	non	300											
23	PHY-1024		49,8	156	1,56	20,46	ivermectine		3200	oui	non	non	oui	non	400	non	non	non
	non	non	oui	non	légère	non	non	non	oui	non	non	non	700	non	non	non	non	non
	non	non	légère	non	non	non	non	non	non	non	48							
								1	non	non	non	non	non	non	non	légère	non	non
	non	non	non	non	non													
24	PHY-1043		67,3	171	1,71	23,02	ivermectine		2000	non	non	non	non	non	900	non	non	non
	non	non	non	non	légère	non	non	non	non	non	non	non	31	non	non	non	non	non
	non	non	légère	non	non	non	non	non	non	non	300							
25	PHY-1045		82,1	159	1,59	32,47	ivermectine		900	non	non	non	non	non	400	non	non	non
	non	non	non	non	légère	non	non	non	non	non	non	non	200	non	non	non	non	non
	non	non	légère	non	non	non	non	non	non	non	2	non	non	non	non	non	non	non
	légère	non	non	non	non	non	non	non	1	non	non	non	non	non	non	non	légère	non
	non	non	non	non	non	non												
26	PHY-1066		54,2	160	1,6	21,17	A400	1000	non	non	non	non	non	700	non	non	non	non
	non	oui	oui	légère	non	non	non	non	non	non	non							

27	PHY-1077		50,4	164	1,64	18,74	ivermectine		1100	oui	oui	non	non	non	400	non	non	non
	non	non	non	non	légère	non	non	non	non	non	non	non	400	non	non	non	non	non
	non	non	légère	non	non	non	non	non	non	non	200	non	non	non	non	non	non	non
	légère	non	non	non	non	non	non	non	100	non	non	non	non	non	non	non	légère	non
	non	non	non	non	non	non												
28	PHY-1105		67,4	170	1,7	23,32	ivermectine		1200	non	non	non	non	non	200	non	non	non
	non	non	oui	non	légère	non	non	non	non	non	oui	non	100	non	non	non	non	non
	non	non	légère	non	non	non	non	non	non	non	2	non	non	non	non	non	non	non
	légère	non	non	non	non	non	non	non										
29	PHY-1111		59,9	160	1,6	23,40	A400	3000	non	non	non	non	non	600	non	non	non	non
	non	oui	non	légère	non	non	non	non	non	non	oui	1200	non	non	non	non	non	non
	non	légère	non	non	non	non	non	non	non	1100	non	non	non	non	non	non	non	légère
	non	non	non	non	non	non	non	200	non	non	non	non	non	non	non	légère	non	non
	non	non	non	non	non													
30	PHY-1112		58,8	159	1,59	23,26	A400	700	oui	non	non	non	oui	300	oui	non	non	non
	oui	oui	oui	légère	non	non	non	non	non	non	non	200	non	non	non	non	non	oui
	oui	légère	non	non	non	non	non	non	non	5	non	non	non	non	non	non	non	légère
	non	non	non	non	non	non	non	200	non	non	non	non	non	non	non	légère	non	non
	non	non	non	non	non													
31	PHY-1129		69,3	173	1,73	23,15	A400	500	non	non	non	non	non	1000	oui	non	non	non
	oui	oui	oui	légère	non	non	non	non	non	non	non	400	non	non	non	non	non	oui
	oui	légère	non	non	non	non	non	non	non	2	non	non	non	non	non	oui	oui	légère
	non	non	non	non	non	non	non	300	non	non	non	non	non	non	non	légère	non	non
	non	non	non	non	non													
32	PHY-1131		77,1	170	1,7	26,68	ivermectine		1600	non	non	non	non	non	100	non	non	non
	non	non	non	non	légère	non	non	non	non	non	non	non	500	non	non	non	non	non
	non	non	légère	non	non	non	non	non	non	non	400	non	non	non	non	non	non	non

légère non non non non non non non 100 non non non non non non non légère non
non non non non non non

33 PHY-1152 70,6 168 1,68 25,01 ivermectine 500 non non non non non 600
100 non non non non non non
non légère non non non non non non non 200 non non non non non non non légère
non non non non non non non 100 non non non non non non non légère non non
non non non non non

34 PHY-1175 72,3 160 1,6 28,24 ivermectine 800 non non non non non 300 non non non
non non non non légère non non non non non non non 300 non non non non non
oui oui légère non non non non non non non 400 non non non non non non non
légère non non non non non non non 6 non non non non non non non légère non
non non non non non non

35 PHY-1193 62,3 167 1,67 22,34 A400 1000 oui non non oui oui 1100 oui non non non
oui oui oui légère non non non non non non non 600 oui non non non oui oui
oui légère non non non non non non non 600 non non non non non oui oui légère
non non non non non non non

36 PHY-1217 54,1 152 1,52 23,42 ivermectine 1600 non non non non non 400 non non non
non non oui oui légère oui non non non non non non 200 non non non non non
oui oui légère non non non non non non non 100 non non non non non oui oui
légère non non non non non non non 200 non non non non non non non légère non
non non non non non non

37 PHY-1222 56,2 159 1,59 22,23 ivermectine 1400 non non non non non 1000 non non non
non non oui oui légère non non non non non non non

38	PHY-1223		60,1	164	1,64	22,35	ivermectine		800	oui	non	non	non	oui	800	oui	non	non
	non	oui	non	non	légère	non	non	non	non	non	non	non	500	oui	non	non	non	oui
	non	non	légère	non	non	non	non	non	non	non	200	oui	non	non	non	oui	non	non
	légère	non	non	non	non	non	non	non	1	non	non	non	non	non	non	non	légère	non
	non	non	non	non	non	non												
39	PHY-1228		64,1	150	1,5	28,49	A400	500	non	non	non	non	non	400	non	non	non	non
	non	non	non	légère	non	non	non	non	non	non	non	17	non	non	non	non	non	non
	non	légère	non	non	non	non	non	non	non	1,00	non	non	non	non	non	non	non	légère
	non	non	non	non	non	non	non	1	non	non	non	non	non	non	non	légère	non	non
	non	non	non	non	non													
40	PHY-1260		52,3	170	1,7	18,10	ivermectine		1700	non	non	non	non	non	900	non	non	non
	non	non	non	non	légère	non	non	non	non	non	non	non	700					
									4	non	non	non	non	non	non	non	légère
	non	non	non	non	non	non	non	5	non	non	non	non	non	non	non	légère	non	non
	non	non	non	non	non													
41	PHY-1274		69,9	179	1,79	21,82	ivermectine		2000	oui	non	non	non	oui	2600	non	non	non
	non	non	oui	oui	légère	non	non	non	non	non	non	non	500	non	non	non	non	non
	oui	oui	légère	non	non	non	non	non	non	non	100	non	non	non	non	non	non	non
	légère	non	non	non	non	non	non	non	100	non	non	non	non	non	non	non	légère	non
	non	non	non	non	non	non												
42	PHY-1288		90	157	1,57	36,51	ivermectine		2300	oui	non	non	oui	oui	1000	oui	non	non
	non	oui	non	non	légère	non	non	non	non	non	non	non	600	oui	non	non	non	oui
	non	non	légère	non	non	non	non	non	non	non	400	non	non	non	non	non	non	non
	légère	non	non	non	non	non	non	non										
43	PHY-1289		52,4	149	1,49	23,60	ivermectine		2100	non	non	non	non	non	400	non	non	non
	non	non	non	non	légère	non	non	non	non	non	non	non	100	non	non	non	non	non
	non	non	légère	non	non	non	non	non	non	non	100	non	non	non	non	non	non	non

	légère	non	non	non	non	non	non	non										
44	PHY-1314		63,3	169	1,69	22,16	A400	1700	oui	non	non	non	oui	800				
											300	non	non	non	non	non	oui	oui
	légère	non	non	non	non	non	non	non	12	non	non	non	non	non	non	non	légère	non
	non	non	non	non	non	non	60											
45	PHY-1316		67,7	166	1,66	24,57	A400	700	non	non	non	non	non	59	non	non	non	non
	non	non	non	légère	non	non	non	non	non	non	non	100	non	non	non	non	non	non
	non	légère	non	non	non	non	non	non	non	60	non	non	non	non	non	non	non	légère
	non	non	non	non	non	non	non	1	non	non	non	non	non	non	non	légère	non	non
	non	non	non	non	non													
46	PHY-1322		70,3	151	1,51	30,83	A400	900	oui	non	non	non	oui	1100	oui	non	non	non
	oui	oui	non	légère	non	non	non	non	non	oui	non	500	oui	non	non	non	oui	non
	non	légère	non	non	non	non	non	oui	non	100	oui	non	non	non	oui	oui	non	légère
	non	non	non	non	oui	non	non	200	non	non	non	non	non	non	non	légère	non	non
	non	non	non	non	non													
47	EBE-003		55	159	1,59	21,76	A400	2200	non	non	non	non	non	2000	non	non	non	non
	non	non	non		non	non	non	non	non	non	non	700	non	non	non	non	non	non
	non		non	non	non	non	non	non	non									
48	EBE-014		64	168	1,68	22,68	A400	1100	non	non	non	non	non	100	non	non	non	non
	non	non	non		non	non	non	non	non	non	non							
