# Supplementary material for: Efficacy and safety of albendazole 400 mg for 30 days compared to single dose of ivermectin in adult patients with low Loa loa microfilaremia: A non-inferiority randomized controlled trial
Source: PLoS Negl Trop Dis. 2025 Jun 20;19(6):e0012383. doi: 10.1371/journal.pntd.0012383 (PMC12212867; doi:10.1371/journal.pntd.0012383)
Supplement: S2 Table — The table defined all variables which are in the dataset of S table. (PDF) [file pntd.0012383.s002.pdf]

code	Signification	modality
numéro PHYLECOG	ID participant	
poids (Kg)	weight in kilogram	
taille (Cm)	height in centimeter	
taille (m)	height in meter	
IMC	body mass index	
traitement administré	administred treatment	"A400=albendazole 400 mg;IVM=ivermectin"
Mf D0	microfilaremia at Day 0 (D0)	
symptome D0	symptom at Day 0 (D0)	"oui=yes; non= no"
oedeme D0	Calabar swelling at Day 0 (D0)	"oui=yes; non= no"
ver dans œil D0	adult worm in the eye at Day 0 (D0)	"oui=yes; non= no"
reptation D0	crawling sensation at Day 0 (D0)	"oui=yes; non= no"
prurit D0	pruritus at Day 0 (D0)	"oui=yes; non= no"
Mf D2	microfilaremia at Day 2 (D2)	
symptome D2	symptom at Day 2 (D2)	"oui=yes; non= no"
oedeme D2	Calabar swelling at Day 2 (D2)	"oui=yes; non= no"
ver dans œil D2	adult worm in the eye at Day 2 (D2)	"oui=yes; non= no"
reptation D2	crawling sensation at Day 2 (D2)	"oui=yes; non= no"
prurit D2	pruritus at Day 2 (D2)	"oui=yes; non= no"
ES D2	Adverse event at Day 2 (D2)	"oui=yes; non= no"
asthenie D2	asthenia at Day 2 (D2)	"oui=yes; non= no"
gravité asthenie D2	gravity of asthenia at Day 2 (D2)	légère=mild
maux de tete D2	headache at Day 2 (D2)	"oui=yes; non= no"
vomissements D2	vomiting at Day 2 (D2)	"oui=yes; non= no"
diarrhee D2	diarrhea at Day 2 (D2)	"oui=yes; non= no"
nausee D2	nausea at Day 2 (D2)	"oui=yes; non= no"
vertige D2	vertigo at Day 2 (D2)	"oui=yes; non= no"
appetit D2	appetite increase at Day 2 (D2)	"oui=yes; non= no"
perte d'appetit D2	appetite reduction at Day 2 (D2)	"oui=yes; non= no"
Mf D7	microfilaremia at Day 7 (D7)	
symptome D7	symptom at Day 7 (D7)	"oui=yes; non= no"

oedeme D7 Calabar swelling at Day 7 (D7) "oui=yes; non= no"

ver dans œil D7 adult worm in the eye at Day 7 (D7) "oui=yes; non= no"

reptation D7 crawling sensation at Day 7 (D7)"oui=yes; non= no"

prurit D7 pruritus at Day 7 (D7) "oui=yes; non= no"

ES D7 Adverse event at Day 7 (D7) "oui=yes; non= no"

asthenie D7 astheniaat Day 7 (D7) "oui=yes; non= no"

gravité asthenie D7 gravity of asthenia at Day 7 (D7)légère=mild

maux de tete D7 headache at Day 7 (D7) "oui=yes; non= no"

vomissements D7 vomiting at Day 7 (D7) "oui=yes; non= no"

diarrhee D7 diarrhea at Day 7 (D7) "oui=yes; non= no"

nausee D7 nausea at Day 7 (D7) "oui=yes; non= no"

vertige D7 vertigo at Day 7 (D7) "oui=yes; non= no"

appetit D7 appetite increase at Day 7 (D7) "oui=yes; non= no"

perte d'appetit 7 appetite reduction at Day 7 (D7)"oui=yes; non= no"

Mf D14 microfilaremia at Day 14 (D14)

symptome D14 symptom at Day 14 (D14) "oui=yes; non= no"

oedeme D14 Calabar swelling at Day 14 (D14) "oui=yes; non= no"

ver dans œil D14 adult worm in the eye at Day 14 (D14) "oui=yes; non= no"

reptation D14 crawling sensation at Day 14 (D14) "oui=yes; non= no"

prurit D14 pruritus at Day 14 (D14)"oui=yes; non= no"

ES D14 Adverse event at Day 14 (D14) "oui=yes; non= no"

asthenie D14 asthenia at Day 14 (D14) "oui=yes; non= no"

gravité asthenie D14 gravity of asthenia at Day 14 (D14) légère=mild

maux de tete D14 headache at Day 14 (D14) "oui=yes; non= no"

vomissements D14 vomiting at Day 14 (D14) "oui=yes; non= no"

diarrhee D14 diarrhea at Day 14 (D14) "oui=yes; non= no"

nausee D14 nausea at Day 14 (D14) "oui=yes; non= no"

vertige D14 vertigo at Day 14 (D14) "oui=yes; non= no"

appetit D14 appetite increase at Day 14 (D14) "oui=yes; non= no"

perte d'appetit 14 appetite reduction at Day 14 (D14) "oui=yes; non= no"

Mf D30 microfilaremia at Day 30 (D30)

symptome D30 symptom at Day 30 (D30) "oui=yes; non= no"

oedeme D30 Calabar swelling at Day 30 (D30) "oui=yes; non= no"

ver dans œil D30 adult worm in the eye at Day 30 (D30) "oui=yes; non= no"

reptation D30 crawling sensation at Day 30 (D30) "oui=yes; non= no"

prurit D30 pruritus at Day 30 (D30) "oui=yes; non= no"

ES D30 Adverse event at Day 30 (D30) "oui=yes; non= no"

asthenie D30 asthenia at Day 30 (D30) "oui=yes; non= no"

gravité asthenie D30 gravity of asthenia at Day 30 (D30) légère=mild

maux de tete D30 headache at Day 30 (D30) "oui=yes; non= no"

vomissements D30 vomiting at Day 30 (D30) "oui=yes; non= no"

diarrhee D30 diarrhea at Day 30 (D30) "oui=yes; non= no"

nausee D30 nausea at Day 30 (D30) "oui=yes; non= no"

vertige D30 vertigo at Day 30 (D30) "oui=yes; non= no"

appetit D30 appetite increase at Day 30 (D30) "oui=yes; non= no"

perte d'appetit 30 appetite reduction at Day 30 (D30) "oui=yes; non= no"
