# Supplementary material for: Efficacy and safety of albendazole 400 mg for 30 days compared to single dose of ivermectin in adult patients with low Loa loa microfilaremia: A non-inferiority randomized controlled trial
Source: PLoS Negl Trop Dis. 2025 Jun 20;19(6):e0012383. doi: 10.1371/journal.pntd.0012383 (PMC12212867; doi:10.1371/journal.pntd.0012383)
Supplement: Attachment — Submitted filename: Responses to PLOS Neglected Tropical Diseases_23_10.docx [file pntd.0012383.s004.docx]

**Responses to PLOS Neglected Tropical Diseases**

**Reviewer's Responses to Questions**

**<b>Methods</b></br></br>**

-Are the objectives of the study clearly articulated with a clear testable hypothesis stated?</br>

-Is the study design appropriate to address the stated objectives?</br>

-Is the population clearly described and appropriate for the hypothesis being tested?</br>

-Is the sample size sufficient to ensure adequate power to address the hypothesis being tested?</br>

-Were correct statistical analysis used to support conclusions?</br>

-Are there concerns about ethical or regulatory requirements being met?</br></br>

**Reviewer #1**: Just a comment regarding samples size, authors didn't mention how they calculate it.

**Authors:** Corrected; as follows:

“Sample size was determined using the formulation of sample size calculation for non inferiority trials [21].

N=((pA*(1-pA)+pB*(1-pB))*((qnorm(1-alpha))+qnorm(1-beta)))/((pA-pB-delta)(pA-pB-delta))

Thus, based on an expected 73% (pB) reduction in microfilaremia with ALB and a 90% (pA) reduction with IVM [22–24], and assuming a first-species risk of 5.0% (alpha) and a second-species risk of 10.0% (beta), with a non-inferiority margin of 20% (delta), it was determined that a minimum of 18 (N) patients per group would be required for this study.”

**Reviewer #2**: The objectives are clearly articulated

The study design was appropriate-

The study population are some how described

The sample size is adequate

There is no ethical concerns

**Reviewer #3**: All the. Methods are clearly described.

**<b>Results</b></br></br>**

-Does the analysis presented match the analysis plan?</br>

-Are the results clearly and completely presented?</br>

-Are the figures (Tables, Images) of sufficient quality for clarity?</br></br>

**Reviewer #1**: yes

**Reviewer #2**: -Does the analysis presented match the analysis plan? : Yes

-Are the results clearly and completely presented?: somehow yes

-Are the figures (Tables, Images) of sufficient quality for clarity?: yes

**Reviewer #3**: The result are compiled in a good way for good understamding.

**<b>Conclusions</b></br></br>**

-Are the conclusions supported by the data presented?</br>

-Are the limitations of analysis clearly described?</br>

-Do the authors discuss how these data can be helpful to advance our understanding of the topic under study?</br>

-Is public health relevance addressed?</br></br>

**Reviewer #1**: yes

**Reviewer #2**:

-Are the limitations of analysis clearly described?: No

**Authors:** “The study limitations are now included into the discussion .

However, the study has some limitations. First, due to the nature of the treatments, double blinding was not feasible. Secondly, its non-inferiority design for the comparison of microfilaremia reduction rates between both treatments, during the first 30 days that correspond to ALB administration duration, did not allow to assess other potential clinical and parasitological endpoints. Further studies with higher sample size and longer follow-up design, such as the assessment of parasite density at 3, 6 months which corresponds to the period of larvae productions by the *Loa loa* female worm, would emphasize the urgent need of validating the proposed ALB protocol. Nevertheless, this trial which assesses the early microfilaridal activity of ALB, provide essential information that were lacking for this molecule, in opposite to IVM..

-Do the authors discuss how these data can be helpful to advance our understanding of the topic under study? Not really

**Authors:** “corrected. Please see Page 18, lines 335-345 DSee lines 326-333.” we have specified that : While IVM treatment exhibited a more pronounced microfilaricidal effect, the difference in risk between the two treatments, as indicated by the associated 95% confidence interval, did not exceed the margin of inferiority. This confirms the non-inferiority of ALB treatment. Moreover, ALB treatment demonstrated an adequate clinical response and exhibited good safety profiles similar to IVM. A 30-day albendazole treatment had a significant microfilaricidal activity and good tolerability in patients with low *Loa loa* microfilaremia. This regimen demonstrated an adequate clinical response and exhibited good safety profiles similar to IVM. Moreover, the daily administration of 400 or 800 mg ALB was found to be safe, even for patients with high microfilaremia. This albendazole regimen would be a promising and affordable treatment option forloiasis in exposed populations from hyperendemic settings of Gabon where access to other drugs is limited. Such protocol would also be considered as one of the recommended treatment lines for the case management of the majority of the exposed populations living in resource limited settings due to its affordable price.

**Reviewer #3**: Appropriate

**<b>Editorial and Data Presentation Modifications?</b></br><br/>**

Use this section for editorial suggestions as well as relatively minor modifications of existing data that would enhance clarity. If the only modifications needed are minor and/or editorial, you may wish to recommend “Minor Revision” or “Accept”.

**Reviewer #1**: Authors need to improve the language they used as well as they should mention how they calculate the sample size and software of data analysis used to obtain the results

**Authors:** Done.

Based on an expected 73% (pB) reduction in microfilaremia with ALB and a 90% (pA) reduction with IVM [22,23,25], and assuming a first-species risk of 5.0% (alpha) and a second-species risk of 10.0% (beta), with a non-inferiority margin of 20% (delta), it was determined by using the following formula [24]

$$N=\frac{(pA*(1-pA)+pB*(1-pB))*((qnorm\left( 1-alpha \right))+qnorm(1-beta))}{(pA-pB-delta)^2}$$

Thus, a minimum of 18 (N) patients per group was required for this study.

R statistical software version 4.3.0 was used for data analysis. See page 6, lines 126-130.

**Reviewer #2**: (No Response)

**Reviewer #3**: Ok

**<b>Summary and General Comments</b></br></br>**

Use this section to provide overall comments, discuss strengths/weaknesses of the study, novelty, significance, general execution and scholarship. You may also include additional comments for the author, including concerns about dual publication, research ethics, or publication ethics. If requesting major revision, please articulate the new experiments that are needed.

**Reviewer #1**: N/A

**Reviewer #2**: The authors of this manuscript entitled “Efficacy and Safety of Albendazole 400 mg for 30 Days in Adult Patients with Loa loa Microfilaremia: A Non-Inferiority Randomized Controlled Trial Compared to Ivermectin “have reported an important NTD like issue. Indeed, up to date there is no clear cutting treatment against Loa loa, a vector born neglected diseases, causing enormously morbidity in endemic areas.

While the manuscript deserves to be published some minors reviews are needed and including:

**Abstract:**

• Authors should add in abstract section, the period (year(s)) where the study was conducted.?

**Authors: “**Corrected as follows (page 2, lines 30): The study was conducted from November 2021 to April , 2022 across […] of northern Gabon.”

• Authors should add in abstract section, the technique(s) used for *Loa loa* diagnostic?

**Authors: “**Corrected as following : **“**Following a questionnaire, venous blood was collected in EDTA tubes, and blood direct examination of 10 µL and 4 ml for the leukoconcentration technique were perfomed for *Loa loa* diagnosis. Of 406 identified microfilaremic case, 48 volunteers were randomized, 21 women and 27 men, their mean age was 51±13 years. Globally, 24 received, daily 400 mg albendazole during 30 days, and 24 others were treated with a single course of 200μg/kg ivermectin. Microfilaremia and adverse event were monitored from D0 to D30. (page 2, lines 31-35)**”**

• How long Albendazole was given before the 30 days followed up?

Authors: Albendazole was given daily for 30 days during which microdilaremia	was monitored.

• Unfortunately the study was not designed for the prevalence, therefore I am wondering why it is so important to report this in the abstract as one of your main finding?

- The data has been removed from the abstract (page 2, lines 35).

• What is the risk difference implied? Should this been proportion difference?

- We found more appropriate to present relative risk rather than proportion difference, as the latter cannot be interpreted without its confidence interval.

• The sample size is missing as well in the abstract section (how many participants were allocated to each group) the gender and age rans and so are missing .

**Authors:** Corrected : 48 volunteers were randomized.

• The abstract is missing the perspective for this important study.

**Authors: “**Done, we have specified perspectives in the abstract : Additional studies with longer follow-up design, would emphasize the urgent need of validating this ALB protocol for the case management of exposed populations. (page 2, lines 43-45)”

**Introduction:**

• Line 80 what this sentence “A significant body of research suggests that loiasis should be regarded as a major health issue” means.

- This sentence can be rewritten in these words: “Although initially considered benign, today, numerous studies suggests that loiasis should be regarded as a major health issue”.

**Material and Methods:**

• Do you have a reference for the 20.2 % of *Loa loa* in the Northern?

- Yes, the study of Jean Paul Akue and al in 2011. Added at the line ()

• Are there any rational for chosen Loa loa <8000 and limited to below 3500?

The majority of infected patients have a microfilaremia below 4,000 mf/mL [3,17,18]. Apart from participants living in onchocerciasis endemic settings, who are at risk of fatal adverse events in case of *Loa loa* microfilaremia and who represent less than 20% of the infected population, there is a need to design appropriate and affordable treatment protocol for the routine management of the other population to reduce loiasis morbidity and transmission.

• The reader may be confused, are the 10 microliter served for thick blood smear and the leucoconcentration technique? Any description of the last?

- The 10 microliters are reserved exclusively for the smear and 4 ml for the leukoconcentration technique as described by Ho Thi Sang and Petithory. (see line 124: Venous blood was collected in an EDTA tube for parasitological diagnosis. A 10 µL blood sample was examined directly, and 4 mL for the leukoconcentration …….)

• The randomization procedure is not clear enough. How participant were selected to each arm is not clear.?

**Authors: “**Corrected, follows (page 6 and 7, lines 138-146)”

A randomization list was prepared by a statistician using a random digit table and different written numbers were provided to the study investigators; these latter were unaware of the contents of the randomization list. The randomization list was provided to an independent pharmacist who was not involved neither on patient selection and inclusion, nor on patient follow-up, or data collection and analysis. For each patient, a number was randomly drawn from the pool of digit provided by the statistician. The pharmacist assigned albendazole or ivermectin treatment to the volunteer based on its corresponding position on the list of randomization.

• Was the trial blinding or open? Please clarify, the choice here is subject of the consequence to the validation of the result.

- The trial was single-blinded for the allocation of patient treatment.

**Results:**

• How were the 48 participants selected from 406 positive participants?

- Among the 406 participants, those with microfilaremia between 500 mf/ml at 3500 mf/ml, who accepted to be included with inclusion criteria were selected for the trial. This corresponded to 48 participants.

• What are the reason to exclude the 21 excluded participants?

- These patients refused to be sampled although they had given their informed consent. Please see the flow chart with the clarification in the corresponding cell.

• On the chart flow the 406 is not added up with 394 +48 please revise the numbers?

**Authors: “**Corrected (page 9), “394” was changed to “358”

• What is the sequence of allocation of study participant?

- The order of allocation within the two groups was 1:1

•	Author may structure better the results section. Having a heading of Efficacy of treatments on the reduction of microfilaremia and sub heading as PPT analysis and ITT analysis.

**Authors: “**Done” the order of titles has been changed as requested

• It is not clear if the assessment of efficacy is post treatment. If so the assessment of those receiving Albendazole will occur 30 days after the 30 days course of treatment. Then 30 days after that of ivermectin

- The objective was to monitored microfilaremia throughout the first 30 days of treatment that corresponded to the duration of albendazole treatment. Early microfilaricidal activity which was observed for IVM was assessed in ALB group.

• Regarding safety, adverse events is vague, would author specify which events are we talking about, the new symptoms or worsen symptoms of existing one?

**Authors:** “Subjective and objectives usual symptoms attributable to loiasis were recorded before and throughout the study. In addition, adverse events that corresponded to new symptoms or worsen existing ones were systematically evaluated throughout the study and categorized by severity (page 7, lines 147-148)” changes have been highlighted in yellow.

**Discussion:**

• Line 264 and 287, we suggested to the author to avoid monthly treatment of albendazole and say “month course”

**Authors: “C**orrected”.

**Reviewer #3:** Overall paper is written in a good way
