# Supplementary material for: Efficacy and safety of albendazole 400 mg for 30 days compared to single dose of ivermectin in adult patients with low Loa loa microfilaremia: A non-inferiority randomized controlled trial
Source: PLoS Negl Trop Dis. 2025 Jun 20;19(6):e0012383. doi: 10.1371/journal.pntd.0012383 (PMC12212867; doi:10.1371/journal.pntd.0012383)
Supplement: Attachment — Submitted filename: Responses to PLOS Neglected Tropical Diseases_26_12_2024.docx [file pntd.0012383.s005.docx]

**Responses to PLOS Neglected Tropical Diseases**

**Reviewer's Responses to Questions**

**<b>Methods</b></br></br>**

-Are the objectives of the study clearly articulated with a clear testable hypothesis stated?</br>

-Is the study design appropriate to address the stated objectives?</br>

-Is the population clearly described and appropriate for the hypothesis being tested?</br>

-Is the sample size sufficient to ensure adequate power to address the hypothesis being tested?</br>

-Were correct statistical analysis used to support conclusions?</br>

-Are there concerns about ethical or regulatory requirements being met?</br></br>

**Reviewer #1**: Yes

**Reviewer #2**: (No Response)

**Reviewer #4**: Yes

**<b>Results</b></br></br>**

-Does the analysis presented match the analysis plan?</br>

-Are the results clearly and completely presented?</br>

-Are the figures (Tables, Images) of sufficient quality for clarity?</br></br>

**Reviewer #1**: Yes

**Reviewer #2**: (No Response)

**Reviewer #4**: Yes

**<b>Conclusions</b></br></br>**

-Are the conclusions supported by the data presented?</br>

-Are the limitations of analysis clearly described?</br>

-Do the authors discuss how these data can be helpful to advance our understanding of the topic under study?</br>

-Is public health relevance addressed?</br></br>

**Reviewer #1**: Yes

**Reviewer #2**: (No Response)

**Reviewer #4**: Yes

**<b>Editorial and Data Presentation Modifications?</b></br><br/>**

Use this section for editorial suggestions as well as relatively minor modifications of existing data that would enhance clarity. If the only modifications needed are minor and/or editorial, you may wish to recommend “Minor Revision” or “Accept”.

**Reviewer #1:** Minor Revision

**Reviewer #2:** (No Response)

**Reviewer #4:** its ok

**<b>Summary and General Comments</b></br></br>**

Use this section to provide overall comments, discuss strengths/weaknesses of the study, novelty, significance, general execution and scholarship. You may also include additional comments for the author, including concerns about dual publication, research ethics, or publication ethics. If requesting major revision, please articulate the new experiments that are needed.

**Reviewer #1:** English language needs to be improved. Moreover, research team should write more in Discussion sector

**Authors: “**English language corrected in the entire manuscript.

The discussion section was improved. Please see below pages 16-19, lines 300-367:

This clinical trial represents the first evaluation of a 30-day ALB treatment for the reduction of low *Loa loa* microfilaremia, a common feature in continuously exposed individuals. ALB treatment regimen was not inferior to a single course of IVM for the reduction of *Loa loa* microfilaremia under the threshold of 500 mf/mL, only one month after the first dose administration.

This study would contribute to evidence-based data supporting tailored loiasis treatment recommendations in highly endemic areas where no consensus exist until now. Low microfilaremia carriage is common in endemic Central Africa settings. In Eepublic of Congo, in Cameroon, and in other studies performed in Gabon, the median microfilaremia rarely reached 1500 mf/mL [17,18,26–28]. The data presented here highlight the high frequency (77.3%; n= 314/406) of low *Loa loa* microfilaremia carriers in a large population (more than 1000 participants) as also reported by Akue 10 years ago, and recently by Veletzky [18,29].

Trials performed in endemic or non endemic areas included either patients with hypermicrofilaremia, or short treatment duration, or sequential treatment administration [13,31,32]. Short or sequential treatment courses, although with high dosage (400 or 800 mg ALB daily) were not associated with a significant reduction rate of parasitaemia, even 12 to 24 months following the start of the treatment [13,32]. Indeed, it is suggested that short course of ALB does not significantly reduce microfilaremia and that longer treatment course with higher dosages has probable more significantly embryotoxic and adulticidal effects [31-33]. Gobbi et *al*. treated *Loa loa* infected patients with 400 mg daily intake during 28 days, followed with a single course of IVM, blood clearance of microfilaremia was obtained in 93% of treated participants within 6 months [34]. In the region of Lambarene, a shorter course of ALB 800 mg daily intake (21 days) followed with a single dose of IVM, or a longer course (35 days) of 800 mg ALB resulted in a significant parasite reduction rate at day 30 (91 to 96%) [14]. A retrospective analysis of different regimen of ALB treatment according to the level of microfilaremia, during the routine management of patients seen at the Department of Parasitology-Mycology-Tropical Medicine (DPMTM), showed a significant reduction or complete recovery rates between 6 to 12 months (M’Bondoukwé et al, submitted). The present trial was designed to assess the efficacy of one of these home-based treatment regimens (a course of 400 mg ALB daily intake during 30 days) following standard procedures of a randomized efficacy trial, thus to provide scientifically evidence-based data.

In both the intention-to-treat (8.6%) and the per protocol (8.1%) analysis, the ALB and IVM treated participants experienced a significant decrease in mean microfilaremia from day 0 to day 30. The 95% confidence interval for these differences observed did not reach or exceed the non-inferiority margin of 20%, according to the CONSORT guidelines; thus, confirming the non-inferiority of a month course of ALB compared to IVM.

In other trials, the observed reduction of *Loa loa* microfilaremia was highly significant at Day 30-35 as recorded among our study participants [14,34]. It is interesting to highlight that in hypermicrofilaremic patients, even after 4 to 6 months following a 35 days of albendazole treatment regimen, there was no significant resurgence of high microfilaremia, but, a higher proportion of cured participants in month 6 to 12 [14]. Thus, we can hypothesize that, in case of low microfilaremia, an acceptable cure or parasite clearance rate would be achieved with our proposed regimen after six to 12 months without an additional treatment. Clinical trials with larger sample size and longer follow-up as well as *in vitro*-evaluations are needed to confirm this hypothesis.

During the follow-up, the number of patients with symptoms decreased, and no patient exhibited any loiasis symptoms by day 30 in each treatment group. This highlights an adequate clinical response to both treatments. However, these findings contrast with the results of the retrospective observational study by Gobbi et al., where 31.3% of patients treated with a single dose of IVM and 50% of those treated with ALB (400 mg/day) were still symptomatic after 30 days of treatment [35].

Less than half of the patients in both treatment groups experienced adverse events during the follow-up period, with asthenia being the most common in both groups. The frequency of these adverse events decreased over subsequent visits, indicating the treatments' safety, all of which were mild. Similar findings have been reported in prior studies using comparable dosages, where no participants experienced serious adverse events [35,36]. Regarding the impact of both treatments on eosinophil counts, there was no statistically significant reduction observed in either treatment group during the follow-up period. These results are consistent with Herrick et al., who found no decrease in eosinophil rates after two weeks of IVM treatment [37]. They also align with Klion et al., who reported no reduction in eosinophil rates after 21 days of ALB treatment [23]. Globally, a significant decrease of eosinophil count is observed between 6 and 24 months [38,39].

This study has some limitations. First, the sample size was small according to the high endemicity of loiasis in Gabon, additional data with larger populations need to be generated. Secondly, a 30-day treatment with 400 mg albendazole per day could be associated with various compliance problems, notably due to the prolonged duration of treatment, which could lead to reduced adherence. While no adherence observance evaluation was performed during our routine practice in the DPMTM, patients, who are usually followed up each 3 months during 6 to 12 months, do not usually complain (data from the DPMTM). Thirdly, ALB semi-annual single dose administration is suspected to be associated with a decrease sensitivity of *Trichuris trichiura* [40]. Nevertheless, there are no data on long treatment course impact. Such treatment would cure intestinal helminth infections and would be associated with a significant reduction of STH transmission in areas with high *Loa loa* and STH co-endemicity such as rural settlements of Gabon [17,41].

Strengths of the study include its randomized design, prospective registration, and comprehensive analysis using both per-protocol and intention-to-treat methods. Furthermore, the present results suggest that a month course of daily 400 mg ALB has acceptable parasitological and clinical efficacy, is well tolerated and safe for the reduction of low microfilaremia. Although a longer follow-up duration is needed, these data are valuable information that would contribute to the development of guidelines and recommendations for the treatment of continuously exposed *Loa loa* infected individuals.

- Changes have been highlighted in yellow.

**Reviewer #2**: The authors respond well to my comments. However, some questions are remaining:

1- How many participants did have more than 3500 microfilariae?

**Authors: “**Among the 406 participants with a *Loa loa* microfilaremia

: 178 had less than 500 mf/ml; 136 had microfilaremia between [500; 3500] mf/ml and 92 had more than 3500 mf/ml. Please see the modified flow chart at the Page 10.

2- Were other participants infected and not randomized been treated? If so, how?

**Authors: “**Yes, other participants infected and not randomized been treated according to medical recommendations. (Page 7, lines 156-157)**”** changes have been highlighted in yellow.

3- How were the 48 participants selected from the 358 infected volunteers?

**Authors: “**There were 406 *Loa loa* microfilaremia volunteers; 270 were not included due to microfilaremia level out of the range of 500-3500 mf/mL. The other 88 had non-inclusion criteria. Please see the modified flow chart below (Page 10).

4- In introduction, the author justifies the advantage for using albendazole to treat Loa loa. Among those, there is a cost of albendazole which was 5$. It is not clear if the 5$ stand for the 30 days course of for one tablet. For the last it will cost 150$ vs 20$ and then Ivermectin seems in that case aTordable. The use of Albendazole instead of Ivermectin seems to be related to the safety rather to the cost. This need to be clarifies.

**Authors: “**5$ is the cost of a box of 50 abendazole tablets of 400 mg. That's more than enough for a monthly treatment “Moreover, one month treatment regimen costs less than 5 USD that is the price for fifty 400 mg tablets.” (Page 5, lines 94-95).**”** Changes have been highlighted in yellow.

5- The non-inferiority finding was not tested. It will be interesting to see if the reduction rate between Albendazole and Ivermectin is not statistically significant.

**Authors: (**page 11, lines 242-243 “The risk difference between the two regimens was 8.1% [95% CI: 16.8 ; -0.6%], and the 95% confidence interval did not exceed the margin of non-inferiority.”and page 12, lines 251-253 “The risk difference between ALB and IVM treatment was 8.4% [95% CI: 16.5 ; 0.7%], and the 95% confidence interval did not exceed the margin of non-inferiority.”). If the margin of non-inferiority is not reach in PP and ITT analysis, there is a non-inferiority between two molecules. In contrary, if the margin of non-inferiority is reached in at least one of the analyses (PP and/or ITT), the non-inferiority is not demonstrated.

Information regarding the comparison between the reduction rates was added:

The microfilaremia reduction rate in ALB group was significantly different with the reduction rate in IVM group (p < 0.001). (Page 11, lines 241-242).

“The reduction rates differed between both treatments (p < 0.001).” (Page 12, lines 251) changes have been highlighted in yellow.

However in the Statistical analysis section the requested analysis was added: Page 9, lines 208-213 was completed the following sentences “The reduction rate of microfilaremia was calculated in percentage using the formula: ((Microfilaremia at D0 -Microfilaremia during follow-up) / Microfilaremia at D0) * 100. According to the CONSORT guideline for non-inferiority studies, the risk difference of the main outcome between the control group (IVM treatment) and the investigational product (ALB) group was determined [26]. It was calculated in per protocol and ITT analysis with its respective 95% confidence interval. An exploratory analysis was performed using a Chi2 test, comparing the observed reduction rates in the per-protocol and the intention-to-treat analysis.”

**Reviewer #4:** This manuscript presents an interesting comparison of the efficacy and safety of Albendazole 400 mg for 30 days versus a single dose of Ivermectin in adult patients with Loa loa microfilaremia. Overall, the authors have successfully incorporated most of the reviewers' suggestions into the revised manuscript.

However, I recommend strengthening the discussion on the public health relevance and implications of the study. Specifically, the manuscript should address the potential challenges related to treatment adherence for a 30-day Albendazole regimen compared to the single-dose Ivermectin treatment, highlighting this as a limitation of the study. Additionally, further elaboration on how these findings can guide health surveillance in endemic regions would enhance the manuscript's impact.

**Authors:** The discussion section was improved. Please see below pages 16-19, lines 300-367:

This clinical trial represents the first evaluation of a 30-day ALB treatment for the reduction of low *Loa loa* microfilaremia, a common feature in continuously exposed individuals. ALB treatment regimen was not inferior to a single course of IVM for the reduction of *Loa loa* microfilaremia under the threshold of 500 mf/mL, only one month after the first dose administration.

This study would contribute to evidence-based data supporting tailored loiasis treatment recommendations in highly endemic areas where no consensus exist until now. Low microfilaremia carriage is common in endemic Central Africa settings. In Eepublic of Congo, in Cameroon, and in other studies performed in Gabon, the median microfilaremia rarely reached 1500 mf/mL [17,18,26–28]. The data presented here highlight the high frequency (77.3%; n= 314/406) of low *Loa loa* microfilaremia carriers in a large population (more than 1000 participants) as also reported by Akue 10 years ago, and recently by Veletzky [18,29].

Trials performed in endemic or non endemic areas included either patients with hypermicrofilaremia, or short treatment duration, or sequential treatment administration [13,31,32]. Short or sequential treatment courses, although with high dosage (400 or 800 mg ALB daily) were not associated with a significant reduction rate of parasitaemia, even 12 to 24 months following the start of the treatment [13,32]. Indeed, it is suggested that short course of ALB does not significantly reduce microfilaremia and that longer treatment course with higher dosages has probable more significantly embryotoxic and adulticidal effects [31-33]. Gobbi et *al*. treated *Loa loa* infected patients with 400 mg daily intake during 28 days, followed with a single course of IVM, blood clearance of microfilaremia was obtained in 93% of treated participants within 6 months [34]. In the region of Lambarene, a shorter course of ALB 800 mg daily intake (21 days) followed with a single dose of IVM, or a longer course (35 days) of 800 mg ALB resulted in a significant parasite reduction rate at day 30 (91 to 96%) [14]. A retrospective analysis of different regimen of ALB treatment according to the level of microfilaremia, during the routine management of patients seen at the Department of Parasitology-Mycology-Tropical Medicine (DPMTM), showed a significant reduction or complete recovery rates between 6 to 12 months (M’Bondoukwé et al, submitted). The present trial was designed to assess the efficacy of one of these home-based treatment regimens (a course of 400 mg ALB daily intake during 30 days) following standard procedures of a randomized efficacy trial, thus to provide scientifically evidence-based data.

In both the intention-to-treat (8.6%) and the per protocol (8.1%) analysis, the ALB and IVM treated participants experienced a significant decrease in mean microfilaremia from day 0 to day 30. The 95% confidence interval for these differences observed did not reach or exceed the non-inferiority margin of 20%, according to the CONSORT guidelines; thus, confirming the non-inferiority of a month course of ALB compared to IVM.

In other trials, the observed reduction of *Loa loa* microfilaremia was highly significant at Day 30-35 as recorded among our study participants [14,34]. It is interesting to highlight that in hypermicrofilaremic patients, even after 4 to 6 months following a 35 days of albendazole treatment regimen, there was no significant resurgence of high microfilaremia, but, a higher proportion of cured participants in month 6 to 12 [14]. Thus, we can hypothesize that, in case of low microfilaremia, an acceptable cure or parasite clearance rate would be achieved with our proposed regimen after six to 12 months without an additional treatment. Clinical trials with larger sample size and longer follow-up as well as *in vitro*-evaluations are needed to confirm this hypothesis.

During the follow-up, the number of patients with symptoms decreased, and no patient exhibited any loiasis symptoms by day 30 in each treatment group. This highlights an adequate clinical response to both treatments. However, these findings contrast with the results of the retrospective observational study by Gobbi et al., where 31.3% of patients treated with a single dose of IVM and 50% of those treated with ALB (400 mg/day) were still symptomatic after 30 days of treatment [35].

Less than half of the patients in both treatment groups experienced adverse events during the follow-up period, with asthenia being the most common in both groups. The frequency of these adverse events decreased over subsequent visits, indicating the treatments' safety, all of which were mild. Similar findings have been reported in prior studies using comparable dosages, where no participants experienced serious adverse events [35,36]. Regarding the impact of both treatments on eosinophil counts, there was no statistically significant reduction observed in either treatment group during the follow-up period. These results are consistent with Herrick et al., who found no decrease in eosinophil rates after two weeks of IVM treatment [37]. They also align with Klion et al., who reported no reduction in eosinophil rates after 21 days of ALB treatment [23]. Globally, a significant decrease of eosinophil count is observed between 6 and 24 months [38,39].

This study has some limitations. First, the sample size was small according to the high endemicity of loiasis in Gabon, additional data with larger populations need to be generated. Secondly, a 30-day treatment with 400 mg albendazole per day could be associated with various compliance problems, notably due to the prolonged duration of treatment, which could lead to reduced adherence. While no adherence observance evaluation was performed during our routine practice in the DPMTM, patients, who are usually followed up each 3 months during 6 to 12 months, do not usually complain (data from the DPMTM). Thirdly, ALB semi-annual single dose administration is suspected to be associated with a decrease sensitivity of *Trichuris trichiura* [40]. Nevertheless, there are no data on long treatment course impact. Such treatment would cure intestinal helminth infections and would be associated with a significant reduction of STH transmission in areas with high *Loa loa* and STH co-endemicity such as rural settlements of Gabon [17,41].

Strengths of the study include its randomized design, prospective registration, and comprehensive analysis using both per-protocol and intention-to-treat methods. Furthermore, the present results suggest that a month course of daily 400 mg ALB has acceptable parasitological and clinical efficacy, is well tolerated and safe for the reduction of low microfilaremia. Although a longer follow-up duration is needed, these data are valuable information that would contribute to the development of guidelines and recommendations for the treatment of continuously exposed *Loa loa* infected individuals.

- Changes have been highlighted in yellow.
