# Supplementary material for: Efficacy and safety of albendazole 400 mg for 30 days compared to single dose of ivermectin in adult patients with low Loa loa microfilaremia: A non-inferiority randomized controlled trial
Source: PLoS Negl Trop Dis. 2025 Jun 20;19(6):e0012383. doi: 10.1371/journal.pntd.0012383 (PMC12212867; doi:10.1371/journal.pntd.0012383)
Supplement: Attachment — Submitted filename: PNTD-D-24-01028R2 RESPONSES TO REVIEWER AND EDITOR COMMENTS.docx [file pntd.0012383.s008.docx]

**Responses to PLOS Neglected Tropical Diseases**

**Responses to Reviewers comments**

**<b>** **Editorial and Data Presentation Modifications</b>**

**Reviewer #1**: English language need to be enhanced.

**Authors: “Done,** we have enhanced English language.**”**

**Reviewer #4**: there is no data to be revised, but I believed that improved appetite is not an adverse reaction.

**Authors:** In the study, we have specified that: An adverse event was any untoward medical event related or unrelated to the study medication, which appeared after the first administration of the drug. Nevertheless, we have removed the increase in appetite as suggested (see Fig.5, page 14).

**Fig. 5. Evolution of clinical adverse events over the 30-day follow-up period.** The histogram shows asthenia (gold bars), diarrhoea (aquamarine bars), headache and/or vertigo (fuchsia bars), and nausea (green bar).

**<b>** **Summary and General Comments</b>**

**Reviewer #1**: It's good paper but English language need to be enhanced.

**Authors: “Done,** we have enhanced English language.**”**

**Reviewer #2**: I am still not convinced with the cost of Albendazole. So to alleviate any doubt and to facilitate public health implementation of this important findings, author should provide the reference including the vendor at national level as well as the manufacturer contacts. Should a patient got 50 tablets of Albendazole at the 5$ in Gabon?

**Authors: “Vendor at national level:** UbiPharm Gabon**, Reference:** Albendazole ubigen 400 mg 50 blisters of 1 tablet**, Manufactured by:** Lincoln Pharmaceutical Ltd **for** Ubithera Pharma PVT.Ltd.**”** (see page 3 line 79).

**Reviewer #4**: The revised version follow the scientific criteria for publication in PLOS Neglected Tropical Diseases. There are minor grammatical errors. Additionally I believed that improved appetite is not an adverse reaction

**Authors:** first, the manuscript was revised for the English language. Then, in the study, we have specified that “An adverse event was any untoward medical event related or unrelated to the study medication, which appeared after the first administration of the drug”. Nevertheless, we have removed the increase in appetite as suggested (see Fig.5, page 14).
